# Limb Muscle Reinnervation with the Nerve-Muscle-Endplate Grafting Technique: An Anatomical Feasibility Study

**DOI:** 10.1155/2021/6009342

**Published:** 2021-12-08

**Authors:** Liancai Mu, Jingming Chen, Jing Li, Stanislaw Sobotka, Themba Nyirenda

**Affiliations:** ^1^Upper Airway Research Laboratory, Hackensack Meridian Health Center for Discovery and Innovation, Nutley, NJ 07110, USA; ^2^Department of Otolaryngology, Icahn School of Medicine at Mount Sinai, New York, NY 10029, USA

## Abstract

**Background:**

Peroneal nerve injuries results in tibialis anterior (TA) muscle paralysis. TA paralysis could cause “foot drop,” a disabling condition that can make walking difficult. As current treatment methods result in poor functional recovery, novel treatment approaches need to be studied. The aim of this study was to explore anatomical feasibility of limb reinnervation with our recently developed nerve-muscle-endplate grafting (NMEG) in the native motor zone (NMZ).

**Methods:**

As the NMEG-NMZ technique involves in nerves and motor endplates (MEPs), the nerve supply patterns and locations of the MEP bands within the gastrocnemius (GM) and TA muscles of rats were investigated using Sihler's stain and whole-mount acetylcholinesterase (AChE) staining, respectively. Five adult rats underwent TA nerve transaction. The denervated TA was reinnervated by transferring an NMEG pedicle from the ipsilateral lateral GM. At the end of a 3-month recovery period, maximal muscle force was measured to document functional recovery.

**Results:**

The results showed that the TA was innervated by the deep peroneal nerve. A single MEP band was located obliquely in the middle of the TA. The GM was composed of two neuromuscular compartments, lateral (GM-l) and medial (GM-m), each of which was innervated by a separate nerve branch derived from the tibial nerve and had a vertically positioned MEP band. The locations of MEP bands in the GM and TA muscles and nerve supply patterns demonstrated that an NMEG pedicle can be harvested from the GM-l and implanted into the NMZ within the TA muscle. The NMEG-NMZ pilot study showed that this technique resulted in optimal muscle force recovery.

**Conclusion:**

NMEG-NMZ surgery is feasible for limb reinnervation. Specifically, the denervated TA caused by peroneal nerve injuries can be reinnervated with a NMEG from the GM-l.

## 1. Introduction

Peripheral nerve injuries (PNIs) to the extremities and resultant muscle paralysis are a major source of chronic disabilities which limit the opportunities to work and diminish quality of life [1]. Although a number of surgical procedures have been used to restore motor function following PNIs [2], the currently available nerve repair surgeries result in poor functional recovery [2–4] due primarily to insufficient axonal regeneration and a failure to reinnervate the denervated motor endplates (MEPs) in the target muscle [5–7]. Therefore, there is a pressing need for new methods to improve outcomes.

We developed a novel surgical technique called the nerve-muscle-endplate grafting (NMEG) technique for muscle reinnervation [8]. The ideal is that a denervated muscle could be reinnervated by transplanting an NMEG pedicle from a neighboring donor muscle. An NMEG pedicle is composed of a donor nerve branch and a block of muscle that contains numerous MEPs and nerve terminals. In our neck muscle model, an NMEG was harvested from sternohyoid muscle and implanted to an MEP-free area in the ipsilateral denervated sternomastoid muscle [8]. As MEP reinnervation of a denervated muscle is critical for motor recovery [6, 7], we modified the procedures by implanting the NMEG pedicle to the native motor zone (NMZ) of the target muscle that contains an MEP band and nerve terminals. This NMEG-NMZ is based on the rationale that denervated MEPs in the NMZ are preferential sites for reinnervation. Studies showed that, after nerve injury and/or direct nerve implantation, regenerating axons preferentially make synaptic contact at the original MEPs [9–15]. Unlike other nerve repair methods, NMEG-NMZ provides an abundant source of nerve terminals that favor axonal regeneration. As the NMEG pedicle is implanted to the NMZ of the target muscle, this facilitates rapid axon-MEP connections. We have demonstrated that NMEG-NMZ results in better functional recovery (82% of the control) [16] than NMEG implantation to an MEP-free area in the target muscle (67%) [8]. However, it remains unknown if the NMEG-NMZ technique is effective for limb reinnervation.

The purpose of this study was to determine the anatomical feasibility of transferring an NMEG from the gastrocnemius muscle (GM) to reinnervate the ipsilateral denervated tibialis anterior (TA) muscle in a rat model.

## 2. Materials and Methods

### 2.1. Animals

In this study, ten hind limbs of adult female Sprague Dawley rats (Charles River Laboratories, MA) were obtained after completion of other experiments. The nerve supply patterns and the locations of MEP bands in the GM and TA muscles were studied. In addition, five rats were used in our pilot study to determine the surgical feasibility and functional outcome. These animal studies were ethically reviewed and approved by the Institutional Animal Care and Use Committee prior to the onset of experiments. All animals were handled in accordance with the *Guide for Care and Use of Laboratory Animals* published by the US National Institutes of Health (NIH Publication no. 85–23, revised 1996).

### 2.2. Sihler's Stain

Five fresh left legs of rats were removed and processed with Sihler's stain, a whole-mount nerve staining technique, to map out branching and distribution patterns of the sciatic nerve and its branches. The details regarding Shiner's stain have been given in our previous publications [17, 18]. In brief, the legs were fixed for 3 weeks in 10% unneutralized formalin; macerated and depigmented for 2 weeks in 3% potassium hydroxide (KOH) solution; decalcified for 2 week in Sihler's solution I (one part glacial acetic acid, one part glycerin, and six parts 1% aqueous chloral hydrate) with several changes; stained for 3 weeks in Sihler's solution II (one part stock Ehrlich's hematoxylin, one part glycerin, and six parts 1% aqueous chloral hydrate); and destained for 3 hr in Sihler's solution I. The legs were washed in running tap water for 1 hr between the aforementioned staining steps. The stained legs were then rinsed for 1 hr in 0.05% lithium carbonate solution to darken the nerves, cleared for 3 days in 50% glycerin, and finally, preserved for 4 weeks before microdissection in 100% glycerin with a few thymol crystals for transparency. After transillumination by a xenon light source (model 610; Karl Storz, Endoscopy-America, Culver City, CA), the stained limb muscles were dissected under a dissecting microscope (TYP 3555110; Wild, Heerbrugg, Switzerland) with 10–30x magnification using microsurgical instruments. The nerves supplying the calf muscles were traced from the main trunk of the sciatic nerve to its major branches and terminations within individual calf muscles. Finally, the dissected specimens were photographed with a Nikon camera (model D5300; Nikon, Japan) under transillumination from a xenon light source (P-Frame A-5A, Taiwan).

### 2.3. Whole-mount AChE Staining

Five entire GM and TA muscles on the left side were removed from rat legs. The muscles were treated with whole-mount AChE staining to locate the MEP band as described in our previous publications [18, 19]. Briefly, the entire TA and GM muscles were fixed for 2 hr in 10% phosphate-buffered formalin; washed in 0.1 M phosphate buffer (PB) at pH 7.4 and pH 6.0 for 15 min in each; incubated in stock solution (cupric sulfate 150 mg, glycerin 190 mg, magnesium chloride 500 mg, maleic acid 900 mg, 4% sodium hydroxide 15 ml, 40% sodium sulfate (anhydrous) 85 ml, and acetylthiocholine iodide 100 mg) at pH 6.0 and 37°C for 2 hr; rinsed in 40% sodium sulfate (anhydrous) for 15 min; washed for 15 min in distilled water (DW); immersed for 15 min in 20% potassium ferricyanide; washed in DW for 60 min; and preserved in 50% glycerin for 3 days. The stained muscles were transilluminated by a xenon light source, dissected under a dissecting microscope (TYP 3555110, Wild), and photographed with a Nikon camera (model D5300; Nikon) under transillumination from a xenon light source (P-Frame A-5A).

### 2.4. Surgical Feasibility Pilot Study

After determining the nerve supply patterns and the location of the MEP bands within the TA and GM muscles, we performed NMEG-NMZ surgery in five rats under general anesthesia as described in our previous publications [8, 16]. First, the left TA was denervated by excising a 10 mm segment of its nerve. Both ends of the nerve were ligated to prevent nerve regeneration. Second, an NMEG pedicle containing a block of muscle (∼8 × 6 × 4 mm), axon terminals, and a MEP band with neuromuscular junctions was harvested from the NMZ of the left lateral GM in continuity with its nerve branch. Third, a muscular defect, with dimensions similar to the NMEG pedicle, was made in the NMZ of the left denervated TA. Finally, the NMEG pedicle was embedded into the TA defect and sutured with 10–0 nylon. At the end of the 3-month recovery period, the maximal muscle force of the TA muscles on both sides was measured as described [8, 16] to document functional recovery.

## 3. Results

### 3.1. Calf Muscles

**Figure 1:**
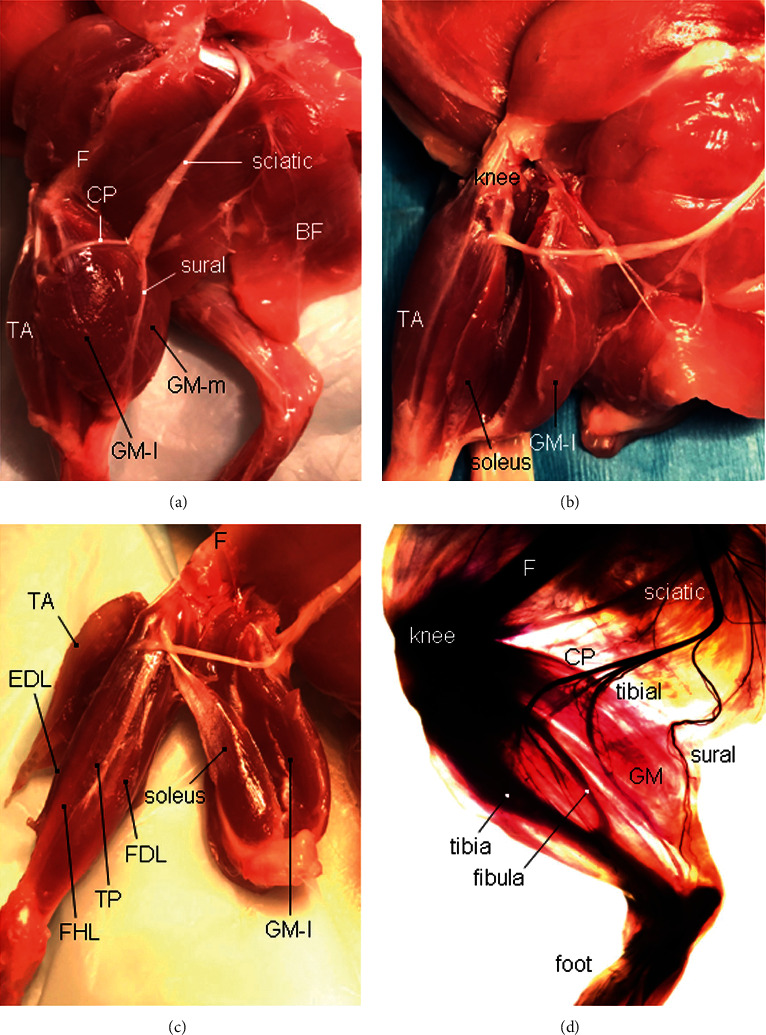
Calf muscles and their innervation of the rat left hind limb. (a) The calf muscle group of the left hind limb is exposed after the biceps femoris (BF) is reflected laterally. CP, common peroneal nerve; F, femur; TA, tibialis anterior muscle; GM-l, lateral compartment of the GM; and GM-m, medial compartment of the GM. (b) Left GM is detached and separated from the soleus muscle. (c) The separated calf muscles, including GM, soleus, flexor digitorum longus (FDL), flexor hallucis longus (FHL), and tibialis posterior (TP) muscle innervated by the tibial nerve, as well as the TA and extensor digitorum longus (EDL) innervated by the deep peroneal nerve. F, femur. (d) A rat left hind limb processed with Sihler's stain without microdissection, showing anatomical relationships among calf muscles, innervating nerves, and bone structures.

Figure 1 shows the calf muscles and major branches of the sciatic nerve in the rat hind limb. The calf muscles include GM, soleus, flexor hallucis longus (FHL), flexor digitorum longus (FDL), tibialis posterior (TP), TA, and extensor digitorum longus (EDL). The GM is composed of two neuromuscular compartments (NMCs), lateral (GM-l) and medial (GM-m).

The TA is a fusiform muscle located in the anterior part of the leg. It arises from the lateral tibia, and its tendon inserts on the medial foot. Along with fibularis tertius, EDL and EHL, it comprises the anterior (extensor) compartment of the leg. TA lies medial to EDL, which makes it the most medial muscle in the anterior compartment of the leg.

### 3.2. Branching and Distribution of Sciatic Nerve

**Figure 2:**
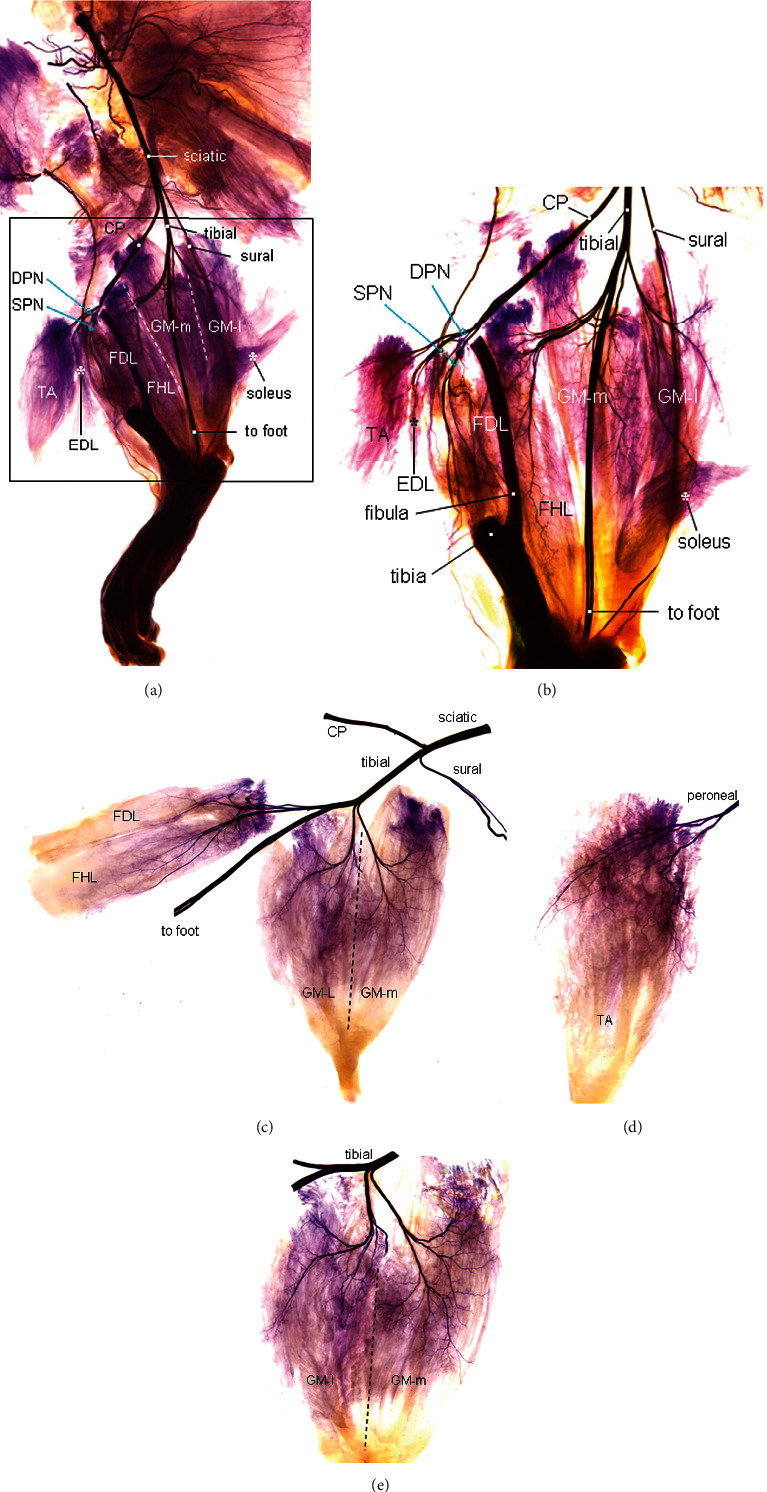
A Sihler's stained rat hind limb, showing the major branches of the sciatic nerve and their distribution in the calf muscles. (a) Low-power view of the Sihler's stained left hind limb after microdissection, 4x. The sciatic nerve is divided into three major branches: CP, tibial, and sural (sensory). The peroneal nerve gives off branches to innervate TA and EDL. The tibial nerve gives off branches to supply the GM, soleus, FHL, FDL, and muscles in the foot. The GM-l and GM-m are outlined by white dotted lines. (b) Magnification of the boxed region in A showing the intramuscular branching and distribution patterns of the peroneal and tibial nerves. The upper part of the tibia was removed. The deep peroneal nerve (DPN) supplies branches to the TA and EDL. The tibial nerve gives off three branches, first branch to the GM, second branch to the foot, and third branch to the FHL and FDL. Also, the GM nerve branch gives off two secondary branches, one innervating the GM-l and soleus and another supplying the GM-m. SPN, superficial peroneal nerve, 12x. (c–e) High-power view of the branching of the sciatic nerve and intramuscular innervation of the tibial and peroneal nerves, 16x.

Sihler's stain (Figures 1(d) and 2) showed that the sciatic nerve is divided into three major branches: common peroneal nerve (CP), tibial nerve, and sural nerve (sensory). The CP winds around the neck of the fibula and divides into a superficial and a deep branch. The deep peroneal nerve (DPN) innervates the TA and EDL in the anterior compartment of the leg.

The tibial nerve innerves the GM, soleus, FHL, FDL, and muscles in the foot (Figure 2). Specifically, tibial nerve gives off three branches, the first branch (the thinnest one) to the GM and soleus, the second branch (the thickest one) to the foot, and the third branch to the FHL and FDL. GM-l and GM-m are innervated by separate nerve branches derived from the tibial nerve. The nerve branch to the GM-l gives off a branch to innervate the soleus muscle (Figures 2(a) and 2(b)).

### 3.3. MEP Bands within the TA and GM Muscles

**Figure 3:**
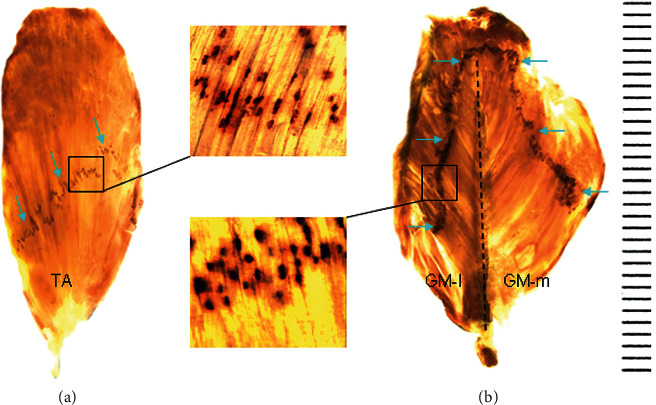
Locations of the MEP bands within the TA and GM as demonstrated by whole-mount AChE staining. (a) The MEP band (arrows) with numerous neuromuscular junctions (black dots) within the TA is located obliquely in the middle of the muscle. (b) Each of the GM-l and GM-m compartments in the GM has its own MEP band (arrows). The vertical dished line in the GM indicates midline.

The MEP band is formed by numerous neuromuscular junctions.  Figure 3 shows the MEP bands within the TA and GM muscles. The MEP band within the TA is located obliquely in the middle of the muscle (Figure 3(a)). In the GM, each of the GM-l and GM-m compartments has its own MEP band which is vertically located (Figure 3(b)).

### 3.4. Surgical Feasibility of NMEG-NMZ in Limb Reinnervation

**Figure 4:**
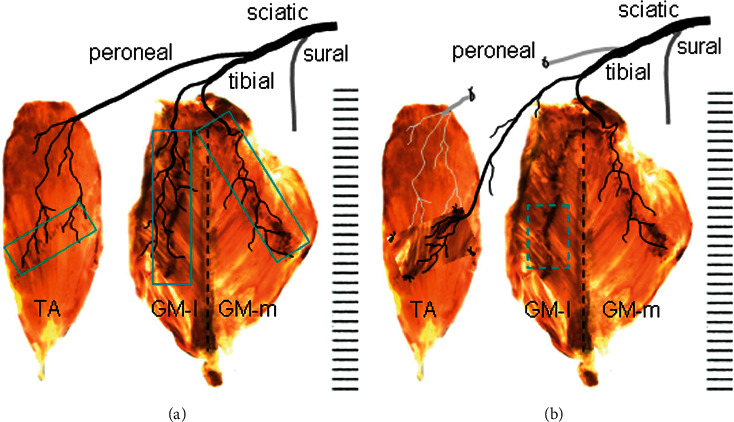
(a) Native motor zones (NMZs) within the TA and GM muscles (boxed regions) as demonstrated by Sihler's stain and AChE staining. (b) TA denervation and NMEG-NMZ transplantation. The TA is denervated by resecting a segment of the peroneal nerve. The denervated TA is treated with the NMEG-NMZ technique. An NMEG pedicle with a nerve branch is harvested from the NMZ of the GM-l (boxed region) and implanted to the NMZ of the denervated TA muscle.

The NMZs within the GM and TA muscles were delineated based on the locations of MEP bands and their innervating nerve terminals (Figure 4(a)). Our NMEG-NMZ pilot study showed that an NMEG pedicle can be harvested from the NMZ of the GM-l and transplanted to the NMZ of the TA (Figure 4(b)). In the rats with NMEG-NMZ surgery (*n* = 5), the average muscle force of the reinnervated TA recovered up to 81% of the contralateral control. These findings suggest that if the TA is denervated following peroneal nerve injury, the NMEG-NMZ technique could be an option to treat “foot drop” caused by TA paralysis.

## 4. Discussion

We investigated the branching and distribution of the sciatic nerve and NMZs within the TA and GM muscles in the rat. This anatomical study on the nerve supply patterns and locations of MEP bands in the TA and GM muscles allows us to identify their NMZs for NMEG-NMZ surgery. Since the GM-l lies adjacent to the TA, an NMEG pedicle from the NMZ of the GM-l could reach to the NMZ of the TA without difficulty. Our pilot study showed that NMEG-NMZ resulted in promising functional recovery three months after limb muscle reinnervation.

TA is the dorsiflexor of the foot and plays a critical role in walking. Paralysis of the TA caused by CP or DPN injuries or lesions results in foot drop, a disabling condition that can make walking difficult and lead to frequent falls.

Traditional treatment modalities include use of an ankle-foot orthosis, tendon surgery, and nerve repair. Tendon transfer surgery is often used to treat foot drop with mixed results [20, 21]. For example, all or a part of the healthy posterior tibial tendon is transferred to the dorsum of the foot for restoring foot dorsiflexion. However, the foot drop tendon transfer surgery results in weak ankle dorsiflexion [22].

Nerve repair [23], nerve grafting [24], and nerve transfer [25–28] are commonly used to manage sciatic and peroneal nerve injuries and lesions. Unfortunately, 64% of repair and grafting of the sciatic nerve [29] and 46–54% of the common peroneal nerve palsies [23, 24, 29] fail to restore functional dorsiflexion. Nerve transfer procedures such as a tibial nerve branch to the deep peroneal nerve [26–28] or a bundle of nerves supplying the soleus and lateral GM to the deep peroneal nerve [25] have been used to treat TA paralysis after peroneal and/or sciatic nerve injuries, which have had mixed results. Therefore, there is a great need to develop new approaches for foot drop treatment.

Poor motor recovery after PNIs and nerve repair is due primarily to insufficient axonal regeneration and failure to reinnervate the denervated MEPs in the target muscle. In response to this, we developed the NMEG. NMEG-NMZ is a recently developed novel surgical technique that targets NMZ for rapid MEP reinnervation, thereby leading to favorable functional recovery. Transplanting an NMEG from GM-l to the NMZ of the TA muscle is anatomically and surgically feasible and could offer several advantages to current treatment options. First, NMEG-NMZ provides an abundant source of nerve terminals that favor axonal regeneration. Second, as an NMEG pedicle is implanted directly to the MEP zone, NMEG-NMZ physically shortens regeneration distances and favors rapid axon-MEP connections. Finally, NMEG has ample pedicle-recipient muscle interfaces, which provide enough space for axonal regeneration at multiple points in the implanted NMEG pedicle and grow across the interfaces to reach the target.

This study showed that transferring an NMEG pedicle from GM-l to the NMZ of the TA can be used to treat TA paralysis caused by CP or DPN injuries. Further experimental studies are needed to evaluate the efficacy of the NMEG-NMZ technique for limb muscle reinnervation.

## Data Availability

All data of this study are available upon request from the first author Liancai Mu.
